# The usability of bedside ultrasound in nursing practice for critically ill patients

**DOI:** 10.1590/0034-7167-2024-0296

**Published:** 2025-04-28

**Authors:** Eduarda Cristina Galon, Denis Fernandes da Silva Ribeiro, Marielli Terassi

**Affiliations:** I Universidade Federal do Paraná, Hospital de Clínicas. Curitiba, Paraná, Brazil

**Keywords:** Ultrasonography, Ultrasonography, Interventional, Nurses, Critical Care, Emergencies, Ultrasonografía, Ultrasonografía Intervencional, Enfermeras y Enfermeros, Cuidados Críticos, Urgencias Médicas

## Abstract

**Objective:**

To describe the usability of bedside ultrasound (POCUS) as perceived by nurses working in emergency, urgent care, and intensive care units.

**Methods:**

We conducted an exploratory study using a mixed-methods approach with nurses in the critical care sectors of a university hospital in Paraná, Brazil. Data collection involved mixed questionnaires and semi structured interviews. We analyzed the data through descriptive statistics and thematic content analysis.

**Results:**

Nurses utilized POCUS for procedures such as venous and arterial punctures, bladder assessments and procedures, cardiovascular assessments, and gastrointestinal evaluations and procedures. Perceptions of usability were organized into thematic categories: Qualification for POCUS certification, Impact of the tool on nursing practice, and Use of the equipment in daily activities.

**Conclusions:**

POCUS was regarded as a significant tool for nursing care, enhancing interventions, clinical decision-making, and professional autonomy.

## RESUMEN

**Objetivo:**

Describir la usabilidad de la ecografía a pie de cama según la percepción de enfermeros en unidades de urgencia, emergencia y cuidados intensivos.

**Métodos:**

Investigación exploratoria de enfoque cuantitativo y cualitativo con enfermeros de sectores críticos de un hospital universitario en Paraná, Brasil. La recolección de datos se realizó mediante cuestionarios mixtos y entrevistas semiestructuradas, analizándose mediante estadística descriptiva y análisis temático de contenido.

**Resultados:**

Los enfermeros empleaban POCUS en procedimientos como punción venosa y arterial, evaluaciones y procedimientos vesicales, evaluación cardiovascular y procedimientos gastroenterales. Las percepciones sobre la usabilidad se agruparon en categorías: Calificación para la certificación en POCUS, Impacto de la herramienta en la práctica de enfermería y Uso del equipo en actividades diarias.

**Consideraciones finales:**

POCUS se percibió como una herramienta significativa para el cuidado de enfermería, optimizando intervenciones, decisiones y la autonomía profesional del enfermero.

## INTRODUCTION

Point-of-care ultrasound (POCUS) is increasingly utilized by healthcare professionals in hospital settings, given its broad applicability in clinical practice^(1-4)^. This non-invasive and cost effective imaging tool^(2,3)^ allows real-time visualization of organs and structures, enhancing clinical decision-making based on the patient’s current condition.

POCUS has earned the designation “the new stethoscope”^(2,5)^, particularly for its ability to assist in physical examination, assess structures, and guide interventions according to the patient’s clinical status^(6)^. This guidance for interventions distinguishes it from diagnostic ultrasound, which is limited to medical specialists and focuses on diagnosing diseases and health conditions.

With technological advancements, ultrasound devices have become increasingly compact and portable, ranging from traditional machines to wireless probes/transducers that stream real-time images to mobile device screens. This evolution has expanded the use of ultrasound beyond medicine, now encompassing other health professions, including nursing^(7,8)^.

In Brazil, the Federal Nursing Council provides technical support for nurses to use ultrasound under Resolution COFEN No. 679/2021, which approves and regulates bedside and pre hospital ultrasound by nurses^(9)^. With bedside ultrasound, nurses can enhance physical exams, reduce adverse events during procedures^(4)^, and optimize the use of material resources.

Specifically, in Brazilian intensive care, emergency, and urgent care units, the use of POCUS by nurses is gaining traction, given the need for rapid, accurate decisions and for supporting procedures with safety and efficiency. However, we hypothesize that POCUS usability, defined here as its ease of use, depends on factors affecting its operability^(4)^, which can be identified through user perceptions.

The following questions guide this research: How do bedside nurses in emergency, urgent care, and intensive care units perceive POCUS in their daily practice? What factors influence and impact POCUS use in their settings? What interventions are supported by POCUS, according to these nurses?

The relevance of this study stems from the limited exploration and analysis of factors affecting POCUS usability and of nurses’ perceptions within critical care. Thus, these findings are crucial for contributing data to the literature, particularly given the novelty of this approach in Brazilian and Latin American contexts.

## OBJECTIVE

To describe the usability of bedside ultrasound (POCUS) as perceived by nurses in emergency, urgent care, and intensive care units and to identify the interventions supported by POCUS in critical care settings based on nurses’ perspectives.

## METHODS

### Ethical aspects

The institution’s Research Ethics Committee approved the study. All participants were volunteers and signed the Informed Consent Form (ICF). Participants were coded using the letter N for “nurse,” followed by a randomly assigned Arabic numeral from 1 to 10.

### Study design, period, and setting

This study employed an exploratory, mixed-methods approach. The quantitative phase adhered to STROBE guidelines, while the qualitative phase followed the COREQ criteria of the EQUATOR network. Data collection occurred between March and April 2023 in a large tertiary university hospital in Paraná, Brazil. Four adult critical care sectors were included: three intensive care units and one emergency and urgent care department.

### Theoretical and methodological framework

This study is grounded in the theoretical-methodological framework of the sociology of professions, as proposed by Durkheim^(10)^, and the hermeneutic-dialectic method developed by Minayo^(11)^. The sociology of professions framework offers insights into professional roles and healthcare’s social division of labor. The hermeneutic-dialectic method aids in interpreting the social, historical, and political contexts relevant to the study topic.

### Population, sampling, and selection criteria

The sampling technique followed an intentional/convenience-based non-probabilistic method. Participants who met the inclusion criteria were invited to participate, and those individuals then referred others known for their expertise in POCUS, following the snowball sampling technique.

The inclusion criteria were 1) being a nurse, 2) working in the selected critical care sectors during data collection, and 3) having certification in ultrasound/POCUS. Qualified professionals were those with specific training in ultrasonography as defined by COFEN Resolution No. 679/2021. Exclusion criteria included 1) nurses on vacation or leave during data collection and 2) nurses who rescheduled their data collection interview more than twice.

### Data Collection and Organization Procedures

Data collection occurred in two sequential stages. In the first stage, we administered a questionnaire comprising both openand closed-ended questions about sociodemographic information, education level, years of experience, and procedures supported by POCUS. We organized these data into a Microsoft Office Excel database for subsequent statistical analysis.

In the second stage, we conducted individual semi-structured interviews in a reserved setting at the workplace, scheduled according to participants’ availability and preferences. We made audio recordings using an offline native smartphone recorder with participants’ prior consent and transcribed them into Microsoft Office Word text files for analysis. The data were stored in encrypted offline folders to ensure protection and accessibility exclusively to the research team. Participants’ identities were coded with unique pseudonyms.

### Data Analysis

We analyzed the data according to their nature. Quantitative data underwent statistical analysis, including measures of central tendency (mean, median, absolute numbers, and percentages) calculated in Microsoft Office Excel and measures of dispersion (standard deviation, range, and variance) calculated in IBM SPSS Statistics.

Qualitative data were analyzed through thematic content analysis, following Bardin’s framework^(12)^. This method includes three phases: pre-analysis (floating reading of the material), material exploration (defining emerging categories and classifying discourse segments), and treatment of results (analyzing and interpreting findings).

## RESULTS

### Participant profile

The study included ten nurses who work directly with critically ill patients and integrate POCUS into their routine clinical practice. Table 1 provides an overview of the participants’ profiles.

**Table 1:** Profile of participating nurses. Curitiba, Paraná, Brazil, May/2024

Variables	Total
	n = 10
Gender	
Male	60%
Female	40%
Age	
Mean	36,2 years
Median	36 years
Range	31 to 48 years
Highest level of education	
Specialization	70%
Master’s	30%
Years of professional experience	
Mean	12,5 years
Median	10 years
Mode	10 years
Range	7 to 26 years
Years of experience in critical care	
Mean	6.1 years
Median	2.5 years
Mode	2 years
Range	1 to 26 years
Years of POCUS certification	
Mean	9.1 months
Median	8.5 months
Mode	8 months
Range	2 to 18 months

### Procedures supported by POCUS

The data indicate that all nurses (100%) used POCUS for peripheral venous puncture (for blood collection or access), arterial puncture (for blood collection or access), and bladder assessments or related procedures (including bladder evaluations and catheter-related interventions). Additional nursing procedures supported by POCUS included cardiovascular assessments, gastrointestinal evaluations and procedures (such as positioning nasogastric and nasoenteric tubes and assessing fecal impaction), lung assessments, verification of orotracheal tube placement, and intracranial pressure assessment (through optic nerve sheath diameter measurement).

### Categories related to POCUS use in the study setting

Analysis of nurses’ perceptions of POCUS usability revealed three thematic categories: 1) qualification for POCUS certification, 2) impact of the tool on nursing practice, and 3) use of the equipment in nurses’ daily activities. These categories, their intermediate subcategories, and corresponding discourse excerpts are presented in Chart 1.

**Chart 1:** Emerging categories in the empirical data derived from nurses’ perceptions of POCUS usability. Curitiba, Paraná, Brazil. May/2024

Thematic categories	Intermediate categories	Meaning units/ Discursive fragments
Qualification for POCUS certification	Motivation as a starting point for POCUS certification	*I have always liked taking courses that would improve my care skills, and here at the hospital, I saw that I had this opportunity. So I found it interesting, learned to use* [POCUS], *and went deeper into it.* (E7) *My main motivations for taking the course were to improve the quality of care and to facilitate procedures that, with the conventional method* [without POCUS], *would either be impossible or have a low success rate. So* [I took the POCUS course] *not only to guide procedures but also to gain more patient data from an assessment perspective and to complement the physical examination.* (E5) *Since this is a Unified Health System (SUS)-qualified hospital* [teaching hospital], *where we have excellent teaching, opportunities, and access to technology* […] *I just wanted an additional skill to feel more secure and to improve care quality.* […] *I sought the training and could bring that back to my work by having more expertise in technology that impacts patient care.* (E9)
	Workplace demands as drivers for POCUS certification	*I learned with the medical team. I used to see them performing* [bedside ultrasound] *and started getting interested. So, to avoid always asking them to do it,* […] *they explained it to me, and I learned to do it too* [since 2019]. (E1) *Today, in the intensive care setting, it’s essential! A nurse must have expertise in what they’re doing. A nurse attentive to nursing care quality must seek all technological improvements, and POCUS is part of that.* (E9)
	Availability of technological resources as an enabler	[…] *with the device available, you end up feeling motivated to improve.* (E8) *One motivating factor was that, if I have the technology in this teaching-driven hospital environment, why not use it?* (E9)
Impact of the tool on nursing practice	Contributions to patient safety	*After the course, my care approach changed, reducing the risk of adverse events-such as cases of multiple* [vascular] *puncture attempts-because ultrasound makes it easier.* (E10) *I think we can see more. Not only focusing on the patient’s clinical presentation, signs, and symptoms but also having an additional tool, which is imaging.* (E4) *Critically ill patients often develop edema and anasarca, making it more difficult to access the vein or artery. With POCUS, we can better visualize where and how that vessel is passing. This way, you can access* [the vein or artery] *more quickly and easily.* (E7)
	Reduction of care costs	*Material savings are an issue. For example, with ultrasound, you avoid multiple failed puncture attempts, as you succeed on the first try, significantly reducing unit expenses.* (E7) […] [Ultrasound] *causes less distress for the patient and saves costs for the institution since you’re not using multiple catheters due to repeated puncture attempts.* (E2) *The technical team even encourages using ultrasound when there’s a difficult puncture. They’ll already ask for the ultrasound-guided approach because it reduces material waste*. (E8)
	Decision-making	*Ultrasound is essential for supporting my clinical practice, enabling me to reason, decide, suggest, and choose one course of action over another. Nowadays, I can’t imagine working in the ICU without an ultrasound* […] *It’s a tool that all nurses should be able to use.* (E1) *Ultrasound is a tool that elevates the quality of care by enhancing your physical examination and assessment and contributing to a more comprehensive clinical reasoning, thus helping in decision-making and streamlining actions* […] (E3) *The use of bedside POCUS makes nurses’ decisions more assertive, giving them greater confidence when performing certain procedures.* (E9)
	Autonomy	*Since you have more data available to assess the patient’s condition, ultrasound gives you autonomy in this sense. You become grounded in data, evidence, and signs and symptoms, allowing you to evaluate the whole set and arrive at a more precise, objective, and accurate nursing diagnosis. Clearly, the chance of making the right choice is higher. And the more successful you are, the greater your credibility. Autonomy stems from making the right decision because you used various tools and achieved the result more easily and successfully.* (E5)
Use of the equipment in nurses’ daily activities	Facilitators: Availability of POCUS in units, real-time imaging, and no usage cost	[…] *One advantage relates to the availability of the device. We have it in the hospital and have the autonomy and freedom to use it. Although there’s only one* [device per unit], *that’s still significant.* (E4) *Having the device always available is the primary advantage, besides providing real-time imaging* […] *And once the device is in the unit, it has virtually no additional cost for the institution.* (E8)
	Challenges: Resistance from professionals, operator dependency, and team allocation	*The difficulty is more technical, related to device operation and practice. I see my colleague in the unit using it frequently; they’re constantly working with the ultrasound. Sometimes we lack this: the practice and repetition needed to learn and improve.* (E1) […] *There’s a more intrinsic difficulty: it’s up to the nurse to seek out practice. Another challenge arises when the schedule is tight. This reduces my bedside time, which, in turn, limits my opportunities to use POCUS. It’s not about access to technology or permission from management but rather about time for routines and adequate staffing.* (E9) *There’s some degree of bias from other professionals, especially a bit of resistance from medical staff, particularly concerning disagreements in practices after using ultrasound.* (E8) […] *sometimes, there are some doubts from certain professionals; I sense resistance. Sometimes, we’re seen as “soft” nurses who need ultrasound to secure access, which is not the point.* (E6)

## DISCUSSION

Considering the increasing use of POCUS among healthcare professionals in critical care settings^(1,4)^, findings from this study provide insights into the usability of this tool based on nurses’ perceptions, including the profiles of these POCUS users and the interventions this tool supports.

The sample profile closely aligns with findings from three other studies conducted in Brazil^(17-19)^ and internationally^(6)^. Nurses’ POCUS certification time reflects the recent regulatory framework established in Brazil in August 2021^(9)^ and the subsequent availability of certification courses in compliance with these legal guidelines.

The most commonly supported procedures and assessments using POCUS in this study correspond with national and international literature, which highlights its use for vein or artery puncture and cannulation^(1-3,6,12-18,21-28)^, bladder assessments and catheterizations^(3,6,13,19,22,29-32)^, and gastrointestinal evaluations and procedures^(3,33-35)^. Less frequently in the literature, POCUS is mentioned for pulmonary assessments^(36-38)^, orotracheal tube positioning verification^(40)^, and intracranial pressure assessments^(38,41-42)^.

However, bedside ultrasound in healthcare is not limited to the procedures mentioned, as it is considered the “fifth pillar” of physical examination^(4,43)^, especially for adding evidence-based insights and greater accuracy to patient evaluations^(1)^. Participating nurses acknowledge this value as a motivator for pursuing training.

Since Brazilian legislation^(9)^ requires training before using POCUS, certification courses served as the starting point for most participants’ POCUS experience. The motivation for training, coupled with the availability of POCUS and the demands of critical care settings, served as guiding factors for these nurses, integrating new knowledge into daily practices.

In the study setting, as in most Brazilian public hospitals, institutional training in bedside ultrasound is not provided, despite its direct benefits to care quality and patient safety^(3,4,6,13,17,20,29,33)^. As a result, nurses often must fund this training on their own, as illustrated by Participant E8. Without sufficient motivation to gain new knowledge and improve care, it is unlikely that professionals would seek out this certification.

Recognizing the demands of the work environment as a driver for certification, even without institutional support, underscores the need for reorienting institutional continuing education programs. Continuing education tailored to service needs should be a priority for healthcare institutions^(19)^, given its direct impact on care quality^(33)^.

These effects surfaced in participants’ accounts, particularly as they recognized POCUS’s contributions to patient safety. Real-time imaging can guide procedures and complement physical examinations, reducing healthcare costs. POCUS enhances the precision of certain procedures, minimizing repeated attempts. Additionally, it supports decision-making and enhances nurses’ autonomy, allowing rapid responses and decisions in specific clinical situations.

Regarding patient safety, nurses identified several advantages, including the reduction of adverse events, especially harm from multiple blind procedure attempts, decreasing patient discomfort; expanded clinical insights that incorporate imaging alongside other evidence; and improved effectiveness, precision, and speed in care delivery by increasing procedure success rates and decreasing time required.

Different studies affirm POCUS’s role in reducing adverse events^(3-4,17,20)^, such as avoiding injuries to vital anatomical structures during invasive procedures^(1,13,15)^, reducing the need for patient transfers for other imaging tests^(5)^, enhancing accuracy and precision in assessments and procedures^(6-8,13,14,16,18)^, and increasing patient satisfaction^(14,17,18)^. For instance, one study^(4)^ documented a preventable incident in which a young Brazilian mother required hand and wrist amputation following a complication from vascular access in obstetric care.

Participants also noted that POCUS contributes to cost reduction, particularly material expenses. However, its impact on healthcare management extends beyond tangible resources, optimizing human and financial resources^(43)^, which are not always tangible to care professionals. A systematic review^(20)^ showed that ultrasound-guided successful vein cannulation not only reduced hospital material costs but also optimized staff time and availability and directly reduced complications.

In healthcare, the focus on organizational sustainability increasingly involves rationalizing material, human, and financial resources, which are finite and sometimes scarce, particularly in public healthcare institutions. Thus, bedside ultrasound is more than just a technological innovation^(17)^; it supports care management.

For POCUS users, several additional benefits were recognized, notably in decision-making and nurse autonomy. These benefits are linked to an enhanced capacity for clinical reasoning, data-driven decision-making, and the ability to justify care choices. Consequently, professionals can make more precise, assertive, and effective decisions, reinforcing their autonomy within the multidisciplinary team.

A swift and dynamic evaluation is essential for immediate interventions in critical care settings. Thus, incorporating POCUS in these settings enhances structured nursing care’s robustness, safety, and effectiveness^(17)^.

Nonetheless, POCUS use by nurses is contested, given the dominant view of bedside ultrasound as a tool primarily for physicians^(5,43)^. Just as the stethoscope and physical examination are not exclusive to any single profession^(44)^ and are widely practiced across healthcare fields, bedside ultrasound should not be restricted to medicine, especially as research and regulations increasingly demonstrate its benefits across various professions.

The broader use of POCUS reflects a larger discussion on the division of responsibility and authority across healthcare professions and the evolving professional roles in response to technological advances and patient needs. Beyond nursing, other professionals such as physical therapists^(45)^, speech-language pathologists^(46)^, and nutritionists^(47)^ have also begun employing this tool in practice. Given its recent adoption, further research is needed to understand the benefits of POCUS for these professionals’ clinical practices.

Nurses also identified factors that ease and hinder POCUS use in daily practice. The availability of ultrasound devices at the bedside was the most common facilitator, as having the device in the care environment is essential for its use.

The availability of ultrasound devices in hospitals depends on various factors, including institutional policies and resources. As ultrasound interfaces continue to modernize^(43)^ and devices become increasingly portable, their distribution and accessibility will likely to improve across various healthcare settings.

Finally, barriers to POCUS use in this study included resistance from other healthcare professionals, staffing limitations affecting bedside care time, and the need for operators to become proficient in equipment handling, techniques, and configurations.

Multidisciplinary resistance to nurse-led ultrasound use may reflect the medical-centered culture of the study institution. Meanwhile, staffing issues that limit nurses’ bedside care time are also seen as a barrier to POCUS use in another Brazilian study^(17)^. Additionally, the technical challenges noted by participating nurses align with findings from another study^(33)^, particularly regarding image clarity and patient cooperation during assessments.

### Study limitations

This study faced limitations related to its single data collection site and the small sample size, which was determined by the number of nurses certified in POCUS at the institution. Additionally, the recent legislation on POCUS use in Brazil limited available literature for a deeper discussion on the topic.

### Contributions to practice

This study expands knowledge on an advanced nursing practice by demonstrating that POCUS is a valuable tool for nursing care, especially in emergency and intensive care units. The findings highlight specific procedures and assessments that POCUS can support, reinforcing its usability in professional nursing practice.

## FINAL CONSIDERATIONS

Based on the perspectives of nurses working in emergency, urgent care, and intensive care units, this study described POCUS usability and identified interventions supported by this technology in critical care settings. Bedside ultrasound was shown to be a significant innovation with the potential to enhance nursing care.

Participants viewed POCUS as an essential tool for nursing practice in emergency and intensive care units. In these settings, it improves decision-making and autonomy in managing patient care, enabling nurses to conduct assessments before, during, and after interventions.

POCUS was notably used for nursing-related procedures such as peripheral venous access, arterial puncture, and bladder assessments or interventions. Additionally, it supported cardiovascular assessments, gastrointestinal evaluations and procedures, lung assessments, orotracheal tube placement checks, and intracranial pressure evaluations. These findings emphasize its practical usability in daily nursing care.

Given POCUS’s transformative potential for nursing practice, we recommend further studies exploring its usability and effectiveness across various nursing care settings, focusing on contributions to patient safety as documented in clinical records. Institutional and government investment should also be enhanced to broaden nurse training in POCUS use, ensuring its benefits are accessible fully and safely to all.
