# Supplementary material for: The usability of bedside ultrasound in nursing practice for critically ill patients
Source: Rev Bras Enferm. 2025 Apr 28;78(2):e20240296. doi: 10.1590/0034-7167-2024-0296 (PMC12037187; doi:10.1590/0034-7167-2024-0296)
Supplement: Translation

# Translation: A usabilidade do ultrassom à beira-leito na prática assistencial de enfermeiros em pacientes críticos

**DOI:** 10.1590/0034-7167-2024-0296pt

**Authors:** Eduarda Cristina Galon, Denis Fernandes da Silva Ribeiro, Marielli Terassi

**Affiliations:** I Universidade Federal do Paraná, Hospital de Clínicas. Curitiba, Paraná, Brasil

**Keywords:** Ultrassonografia, Ultrassonografia de Intervenção, Enfermeiras e Enfermeiros, Cuidados Críticos, Emergências

## Abstract

**Objetivo:**

Descrever a usabilidade do ultrassom à beira do leito na percepção de enfermeiros atuantes em unidades de urgência, emergência e cuidados intensivos.

**Métodos:**

Pesquisa exploratória de abordagem quantiqualitativa com enfermeiros de setores críticos de um hospital universitário do Paraná, Brasil. A coleta de dados ocorreu por questionários mistos e entrevistas semiestruturadas. Os dados passaram por análise estatística descritiva e análise temática de conteúdo.

**Resultados:**

Os enfermeiros usavam o POCUS para procedimentos como punção venosa e arterial, avaliação e procedimentos vesicais, avaliação cardiovascular e avaliação e realização de procedimentos gastroenterais. As percepções sobre a usabilidade foram reunidas nas categorias temáticas: Qualificação para a habilitação em POCUS, Impactos da ferramenta na prática assistencial e Uso do equipamento nas atividades diárias.

**Considerações finais:**

O POCUS foi percebido como ferramenta significativa para o cuidado de enfermagem, otimizando intervenções, decisões e autonomia profissional do enfermeiro.

## INTRODUÇÃO

A ultrassonografia à beira do leito, o *Point-of-Care Ultrasound* (POCUS), vem sendo cada vez mais utilizada pelos profissionais de saúde no âmbito hospitalar, devido à sua vasta aplicabilidade para a prática clínica^(1-4)^. É um exame de imagem não invasivo e econômico^(2,3)^ que permite a visualização de órgãos e estruturas em tempo real, facilitando a tomada de decisão do profissional diante da situação clínica do paciente.

Ele vem sendo denominado como o novo estetoscópio^(2,5)^, especialmente por possibilitar a avaliação de estruturas, por auxiliar o exame físico e por guiar intervenções em face da situação clínica do paciente^(6)^. Essa condução das intervenções é a principal diferença da ultrassonografia diagnóstica, privativa do profissional médico especialista, cujo objetivo principal é o diagnóstico de doenças e condições de saúde.

Com o avanço no desenvolvimento tecnológico, os dispositivos de ultrassom estão cada vez mais compactos e portáteis e vão desde o aparelho de ultrassom tradicional até mesmo um probe/transdutor com conexão sem fio que transmite imagens em tempo real para a tela de dispositivos móveis. Assim, o uso dessa ferramenta passou a se disseminar para além da medicina, alcançando outras profissões da área da saúde, como a enfermagem^(7,8)^.

No Brasil, o Conselho Federal de Enfermagem respalda tecnicamente os enfermeiros para utilizar a ultrassonografia, mediante a Resolução COFEN Nº 679/2021, que aprova e normatiza a ultrassonografia à beira do leito e no ambiente pré-hospitalar por enfermeiros^(9)^. Com o ultrassom à beira do leito, o enfermeiro consegue incrementar o exame físico, além de realizar procedimentos com a redução dos eventos adversos^(4)^ e otimização do uso de insumos materiais.

Especificamente nas unidades de terapia intensiva e de urgência e emergência do território brasileiro, a utilização do POCUS pelo enfermeiro vem ganhando espaço, considerando a necessidade de decisões rápidas e assertivas e a orientação de procedimentos com segurança e eficácia. Entretanto, parte-se da hipótese de que a usabilidade do POCUS, aqui entendida como capacidade de ser usável, é condicionada por fatores que afetam a sua operabilidade^(4)^, os quais podem ser descritos pela percepção dos seus usuários.

Assim, apresentam-se como questões norteadoras desta pesquisa: Como enfermeiros assistenciais de unidades de urgência, emergência e cuidados intensivos percebem o uso do POCUS em seu cotidiano? Quais são os fatores que condicionam e interferem no uso do POCUS em suas realidades? Quais intervenções são apoiadas pelo uso do POCUS na percepção desses enfermeiros?

Esta pesquisa se justifica pela incipiente exploração/análise dos aspectos que influenciam a usabilidade do POCUS por enfermeiros e das percepções desses profissionais, partindo do recorte dos cuidados críticos. Nesse sentido, os achados tornam-se fundamentais para agregar dados à literatura, considerando o ineditismo da abordagem nos contextos brasileiro e latino-americano.

## OBJETIVO

Descrever a usabilidade do ultrassom à beira do leito (POCUS) na percepção de enfermeiros atuantes em unidades de urgência, emergência e cuidados intensivos; e identificar as intervenções apoiadas pelo POCUS em cenários de cuidados críticos com base na percepção de enfermeiros.

## MÉTODOS

### Aspectos éticos

O Comitê de Ética em Pesquisa da instituição sediadora aprovou a pesquisa. Todos os participantes foram voluntários e assinaram o Termo de Consentimento Livre e Esclarecido. Eles foram codificados recebendo a letra E, inicial de “enfermeiro”, seguida por um número arábico (de 1 a 10) atribuído aleatoriamente.

### Desenho, período e local do estudo

Trata-se de uma pesquisa exploratória de abordagem quantiqualitativa. A etapa quantitativa seguiu as recomendações da diretriz STROBE, e a etapa qualitativa seguiu os Critérios COREQ da rede EQUATOR. Os dados foram coletados entre março e abril de 2023. O cenário do estudo foi um hospital universitário terciário de grande porte no estado do Paraná, Brasil. Foram incluídos quatro setores de cuidados críticos para adultos do referido hospital, sendo três setores de terapia intensiva e um serviço de urgência e emergência.

### Referencial teórico-metodológico

Esta pesquisa está ancorada no referencial teórico-metodológico da sociologia das profissões, na perspectiva de Durkheim^(10)^; e no método hermenêutico-dialético, na perspectiva de Minayo^(11)^. O referencial teórico da sociologia das profissões permite a compreensão sobre as corporações profissionais e divisão social do trabalho na saúde. Já o método hermenêutico-dialético contribui para a interpretação sobre os contextos sociais, históricos e políticos envolvidos com o objeto estudado.

### População, amostragem e critérios de seleção

A técnica de seleção amostral seguiu o método não probabilístico intencional/por conveniência, por meio do qual foram incluídos os profissionais que atendiam aos critérios de seleção. Na sequência, eles indicavam outros que eram referências no uso do POCUS no hospital e que, também, atendiam aos critérios de seleção, configurando o método de amostragem *snowball* (bola de neve).

Para selecionar os participantes, os critérios de inclusão foram 1) ser enfermeiro, 2) atuar nos setores de cuidados críticos estudados durante a coleta de dados e 3) ter habilitação/capacitação em ultrassom/POCUS. Foram considerados profissionais habilitados aqueles que realizaram capacitação específica em ultrassonografia, em consonância com a Resolução COFEN nº 679/2021. Os critérios de exclusão foram 1) profissionais que estavam de férias ou licença de qualquer tipo durante a coleta de dados e 2) profissionais que desmarcaram a entrevista de coleta de dados mais de duas vezes.

### Procedimentos metodológicos: coleta e organização dos dados

A coleta dos dados ocorreu em duas etapas sequenciais. Na primeira etapa, os pesquisadores aplicaram um questionário composto por questões abertas e fechadas sobre os dados sociodemográficos, escolaridade, tempo de formação, tempo de experiência e procedimentos apoiados pelo uso do POCUS. Esses dados foram organizados em banco de dados no Microsoft Office Excel para a posterior análise estatística.

A segunda etapa ocorreu por meio de entrevistas semiestruturadas individuais, realizadas no próprio local de trabalho (em ambiente reservado), com agendamento prévio conforme a preferência e disponibilidade dos participantes. O áudio foi gravado por gravador off-line nativo de smartphone com consentimento prévio de cada participante, para posterior transcrição e análise. As gravações foram transcritas na íntegra em arquivos de texto do Microsoft Office Word. Os dados foram mantidos em pastas off-line criptografadas para assegurar sua proteção, com acesso exclusivo pela equipe de pesquisa. Além disso, a identidade dos participantes foi codificada com um codinome individual.

### Análise dos dados

Os dados foram analisados segundo sua natureza. Os dados quantitativos foram alvo de análise estatística mediante o cálculo de medidas de tendência central (médias, medianas, números absolutos e percentuais) pelo software Microsoft Office Excel; e medidas de dispersão (desvio padrão, amplitude e variância) no software IBM SPSS Statistics.

Já os dados qualitativos passaram por análise temática de conteúdo, na perspectiva de Bardin^(12)^. Foram seguidas as três fases propostas pelo método: pré-análise (leitura flutuante do material), exploração do material (definição das categorias emergentes e classificação dos fragmentos discursivos) e tratamento dos resultados (análise e interpretação dos achados).

## RESULTADOS

### Perfil dos participantes

Participaram do estudo dez enfermeiros que atuavam diretamente com pacientes críticos e utilizavam o POCUS no cotidiano assistencial. A seguir, a Tabela 1 apresenta um panorama do perfil dos participantes.

**Tabela 1:** Perfil dos enfermeiros participantes do estudo. Curitiba, Paraná, Brasil, maio/2024

Variáveis	Total
	n = 10
Gênero	
Masculino	60%
Feminino	40%
Idade	
Média	36,2 anos
Mediana	36 anos
Amplitude	31 a 48 anos
Maior escolaridade	
Especialização	70%
Mestrado	30%
Tempo de atuação profissional	
Média	12,5 anos
Mediana	10 anos
Moda	10 anos
Amplitude	7 a 26 anos
Tempo de experiência em setores críticos	
Média	6,1 anos
Mediana	2,5 anos
Moda	2 anos
Amplitude	1 a 26 anos
Tempo de habilitação em POCUS	
Média	9,1 meses
Mediana	8,5 meses
Moda	8 meses
Amplitude	2 a 18 meses

### Procedimentos apoiados pelo POCUS

Os dados revelam que todos os enfermeiros (100%) utilizavam o POCUS para punção venosa periférica (coleta ou acesso), punção arterial (coleta ou acesso) e avaliação e/ou procedimentos vesicais (avaliação de bexiga ou relacionados a cateteres vesicais). Outros procedimentos de enfermagem apoiados pelo POCUS foram mencionados, sendo eles a avaliação cardiovascular; a avaliação e a realização de procedimentos gastroenterais (incluindo o posicionamento de sondas nasogástricas e nasoenterais e avaliação de fecaloma); a avaliação pulmonar; a verificação de posicionamento de tubo orotraqueal; e a avaliação de pressão intracraniana (pela mensuração da bainha do nervo óptico).

### Categorias relacionadas ao uso do POCUS no cenário estudado

Quanto à análise da percepção dos enfermeiros sobre a usabilidade do POCUS, emergiram três categorias temáticas: 1) Qualificação para a habilitação em POCUS; 2) Impactos da ferramenta na prática assistencial; 3) Uso do equipamento nas atividades diárias de enfermeiros. Tais categorias, suas categorias intermediárias e fragmentos discursivos classificados são apresentados no Quadro 1.

**Quadro 1:** Categorias emergentes no material empírico proveniente das percepções dos enfermeiros participantes sobre a usabilidade do POCUS. Curitiba, Paraná, Brasil, maio/2024

Categorias temáticas	Categorias intermediárias	Unidades de sentido/ Fragmentos discursivos
Qualificação para a habilitação em POCUS	Motivação como ponto de partida para a qualificação em POCUS	*Eu sempre gostei de fazer capacitações que melhorassem a minha assistência; e aqui, no hospital, eu vi que eu tinha essa abertura. Daí achei interessante, aprendi a usar* [o POCUS] *e me aprofundei.* (E7) *Minhas maiores motivações em realizar o curso foram para melhorar a qualidade da assistência e facilitar os procedimentos que, pelo método convencional* [sem o POCUS], *seriam impossíveis ou com baixa taxa de sucesso. Então* [fiz o curso em POCUS] *não só para guiar procedimentos, mas também para obter mais dados dos pacientes, do ponto de vista de avaliação, e para complementar o exame físico.* (E5) *Por ser um hospital de qualificação do SUS* [hospital de ensino], *onde você tem o ensino de excelência, possibilidades e o acesso às tecnologias* [...] *eu queria apenas uma habilidade a mais, para me sentir mais segura e para galgar uma qualidade na assistência.* [...] *Eu busquei a capacitação e pude dar o retorno para o meu trabalho ao ter maior propriedade de uma tecnologia com repercussão na assistência.* (E9)
	Demandas do ambiente de trabalho como indutores para a qualificação em POCUS	*Eu aprendi com a equipe médica. Eu os via fazendo* [ultrassonografia à beira do leito] *e comecei a me interessar. E aí, para eu não ficar toda hora pedindo para eles fazerem* [...] *eles me explicaram e aprendi a fazer também* [desde 2019]. (E1) *Hoje, no cenário de terapia intensiva, é fundamental! O enfermeiro tem que ter expertise naquilo que trabalha. O enfermeiro antenado com a qualificação da assistência de enfermagem tem que buscar todas as melhorias tecnológicas, e o POCUS faz parte disso.* (E9)
	Disponibilidade do recurso tecnológico como impulsionador	[...] *com o aparelho disponível, você acaba se motivando a se aprimorar.* (E8) *Um ponto de estímulo foi que, se eu tenho a tecnologia neste ambiente hospitalar motivado pelo ensino, por que não utilizar?* (E9)
Impactos da ferramenta na prática assistencial	Contribuições à Segurança do Paciente	*Depois do curso, o meu atendimento mudou, diminuindo o risco de eventos adversos - por exemplo, nos casos de múltiplas tentativas de punção* [vascular] *- porque o ultrassom facilita.* (E10) *Eu acho que a gente consegue olhar mais. Não apenas olhar para a clínica do paciente, sinais e sintomas, mas ter um adicional, que é a imagem.* (E4) *No caso dos pacientes críticos, eles comumente desenvolvem edema e anasarca, ficando mais difícil de acessar a veia ou artéria. Com o POCUS, a gente consegue visualizar melhor onde e como aquele vaso está passando. Assim, você consegue acessar* [a veia ou artéria] *em menos tempo e com mais facilidade.* (E7)
	Redução de custos assistenciais	*A economia de material é uma questão. Por exemplo, com o ultrassom você não fica puncionando várias vezes, em tentativas falhas, pois você consegue de primeira e, assim, consegue diminuir consideravelmente os gastos na unidade.* (E7) [...] [O ultrassom] *gera menos sofrimento para o paciente e menos gastos para a instituição, já que você não usa um monte de cateter nos casos de várias tentativas de punção.* (E2) *A equipe técnica até instiga o uso do ultrassom quando se tem uma punção mais difícil. Eles já pedem para puncionar com ultrassom, pois reduz o desperdício de material*. (E8)
	Tomada de decisões	*O ultrassom é fundamental para embasar minha prática assistencial, para argumentar, decidir ou sugerir e optar por uma coisa ou outra. Hoje em dia, já não vejo mais como a gente trabalhar aqui na UTI sem o ultrassom* [...] *é uma ferramenta que todos os enfermeiros deveriam conseguir usar.* (E1) *O ultrassom é uma ferramenta que eleva a qualidade do atendimento, porque amplia o teu exame físico, a tua avaliação e contribui para um raciocínio clínico mais completo, auxiliando na tomada de decisões e encurtamento da atuação* [...] (E3) *A utilização do POCUS à beira do leito tornam mais assertivas as decisões do enfermeiro, pois dá mais confiança para realização de alguns procedimentos.* (E9)
	Autonomia	*Como você tem mais dados disponíveis para fazer um julgamento sobre a situação do paciente, o ultrassom te dá autonomia nesse sentido. Você passa a estar fundamentado em dados, em evidências e em sinais e sintomas, com isso você julga todo esse conjunto e chega a um diagnóstico de enfermagem mais preciso, objetivo e acurado. É evidente que a chance de você acertar é maior. E quanto mais você acerta, mais sucesso você tem e maior a sua credibilidade. A autonomia vem do fato de você tomar a decisão correta, porque utilizou diversos instrumentos e por ter alcançado o resultado com mais facilidade e com maior sucesso.* (E5)
Uso do equipamento nas atividades diárias de enfermeiros	Facilidades: A disponibilidade do POCUS nos setores, o fornecimento de imagem em tempo real e o custo zero para utilização.	[...] *Uma facilidade está relacionada à disponibilidade do aparelho. Nós temos no hospital e temos autonomia e liberdade para utilizar. Apesar de ser só um* [aparelho por setor]*, já é bastante coisa.* (E4) *Ter o aparelho sempre disponível é a principal facilidade, além dele fornecer uma imagem em tempo real* [...] *E, depois que o aparelho já está no setor, ele tem um custo praticamente zero para a instituição.* (E8)
	Dificuldades: Resistência de profissionais, operador dependente e dimensionamento de equipe	*A dificuldade é mais técnica, de operacionalização do aparelho, e relacionada à prática. Eu vejo o meu colega enfermeiro do setor: ele usa muito mais do que a gente, então o tempo todo ele está lá com o ultrassom. Às vezes, nos falta isso: essa prática, essa repetição, para a gente aprender e se aprimorar.* (E1) [...] *Tem uma dificuldade, que é mais intrínseca: é do enfermeiro procurar praticar. Outra dificuldade é quando as escalas estão bastante apertadas. Isso diminui o meu tempo de assistência à beira do leito, o que, por consequência, diminui a minha possibilidade de desfrutar do POCUS. Não é o acesso à tecnologia nem a permissibilidade da chefia, mas, sim, o tempo para as rotinas e dimensionamento de pessoal.* (E9) *Tem algum tipo de preconceito de outros profissionais, principalmente um pouco de resistência da parte médica, sobretudo em questões de divergências de condutas depois da utilização do ultrassom.* (E8) [...] *às vezes, tem alguns questionamentos de alguns profissionais; eu sinto uma resistência. Às vezes, a gente é tido como um enfermeiro “nutella”, que precisa utilizar o ultrassom para garantir um acesso, sendo que não é esse o propósito.* (E6)

## DISCUSSÃO

Considerando o crescente uso do POCUS por profissionais da saúde nos cenários de cuidados críticos^(1,4)^, os achados do presente estudo permitem um panorama sobre a usabilidade da ferramenta na percepção de enfermeiros, perpassando pelo perfil desses usuários do POCUS e pelas intervenções apoiadas por essa ferramenta.

O perfil da amostra participante guarda semelhanças com o encontrado em três outros estudos na realidade brasileira^(13-15)^ e internacional^(6)^. Sobre o tempo de habilitação em POCUS pelos enfermeiros, este decorre da regulamentação recente no Brasil, em agosto de 2021^(9)^, e da subsequente oferta dos cursos de habilitação em conformidade com a legislação.

Os procedimentos e avaliações mais apoiados pelo POCUS no cenário estudado alinham-se com os achados na literatura nacional e internacional, em que aparecem em destaque a punção e a canulação de veias ou artérias^(1-3,6,13,14,16-28)^, a avaliação ou realização de sondagens vesicais^(3,6,14,15,22,29-32)^, bem como a avaliação ou realização de procedimentos em trato gastrointestinal^(3,33-35)^. Com menor frequência na literatura, é mencionado seu uso para avaliações pulmonares^(1,36-39)^, verificação de posicionamento de tubo orotraqueal^(38,39)^ e avaliação de pressão intracraniana^(1,2,40)^.

Entretanto, cabe dizer que a aplicação do ultrassom à beira do leito nos cuidados em saúde não se restringe aos procedimentos citados, já que é considerado um quinto pilar do exame físico^(4,41)^, especialmente por incorporar evidências e maior acurácia à avaliação^(1)^. Tais contribuições desse instrumento são reconhecidas pelos enfermeiros participantes e listadas como indutores da motivação e da busca pela capacitação.

Como a legislação brasileira^(9)^ recomenda a capacitação anterior ao uso da ferramenta, o curso de qualificação foi o ponto de partida da experiência com o POCUS para a maioria da amostra. A motivação para realizar a capacitação e a disponibilidade da ferramenta e das demandas do ambiente de cuidados críticos exercem papel balizador relacionado à capacitação desses profissionais, que aliaram o novo aprendizado às necessidades do dia a dia.

Ressalta-se que, na realidade estudada, assim como na maioria dos hospitais públicos brasileiros, a capacitação em ultrassom à beira do leito não é ofertada institucionalmente, ainda que promova benefícios diretos à qualidade e segurança da assistência^(3-4,6,13,16,20,29,33)^. Isso faz com que os enfermeiros tenham de financiá-la por recursos próprios, tal como visualizado no fragmento discursivo do participante E8. Sem a devida motivação em adquirir conhecimentos e melhorar o cuidado prestado, dificilmente os profissionais buscariam a realização do curso de capacitação.

O reconhecimento das demandas do ambiente de trabalho como indutoras para a capacitação, mesmo sem apoio institucional, remonta à necessidade de reorientação das ações institucionais de educação continuada. A educação continuada com foco nas necessidades e demandas dos serviços deve ser um compromisso das instituições de saúde^(15)^, considerando os reflexos diretos ao cuidado^(33)^.

Esses efeitos emergem no discurso dos participantes, especialmente ao reconhecerem as contribuições do POCUS para a segurança do paciente. É possível agregar a imagem em tempo real para guiar procedimentos e complementar o exame físico, com redução de custos assistenciais. O profissional conta com maior assertividade, diminuindo as tentativas de alguns procedimentos. Além disso, a ferramenta auxilia na tomada de decisões e autonomia do enfermeiro, possibilitando responder e decidir rapidamente qual a conduta a ser tomada diante de determinada situação.

Na perspectiva das contribuições do POCUS para a Segurança do Paciente, as percepções dos enfermeiros giraram em torno dos benefícios à redução de eventos adversos, especialmente: danos decorrentes de múltiplas tentativas de procedimentos às cegas, com a diminuição dos desconforto do paciente; ampliação da clínica, que passa a integrar a imagem junto com as demais evidências; ampliação da resolutividade, assertividade e celeridade do cuidado, ao incrementar as chances de sucesso em procedimentos e diminuir sua duração.

Diferentes estudos reconhecem o papel do POCUS na redução dos eventos adversos^(3,4,13,20)^, ao evitar acidentes envolvendo estruturas anatômicas vitais durante procedimentos invasivos^(1,16,18)^; ao otimizar a necessidade de transportes para realizar outros exames de imagem^(5)^; ao incrementar a acurácia, a precisão e a assertividade da avaliação e de procedimentos realizados pelos profissionais^(6-8,14,16,17,19)^; e ao aumentar a satisfação dos pacientes^(13,14,17)^. Cabe citar o exemplo de incidente evitável documentado em estudo^(4)^, em que uma jovem parturiente brasileira teve a mão e o punho amputados após complicação ocasionada pelo acesso vascular com fins obstétricos.

A redução dos custos assistenciais por meio do POCUS também foi um aspecto percebido, especificamente quanto aos gastos materiais. Todavia, tais repercussões na gestão do cuidado não se restringem somente aos recursos materiais, uma vez que se expandem para a otimização de recursos humanos e financeiros^(41)^, nem sempre tangíveis aos profissionais da assistência. Em revisão sistemática^(20)^, ficou demonstrado que a canulação bem-sucedida de veias pelo ultrassom à beira do leito foi capaz não só de reduzir os custos hospitalares com insumos materiais, mas também de otimizar o tempo e a disponibilidade da equipe assistencial, bem como reduzir diretamente as complicações.

Nos cuidados em saúde, o debate sobre a sustentabilidade organizacional é cada vez mais frequente e envolve a racionalização dos recursos materiais, humanos e financeiros. São recursos finitos e, por vezes, escassos, especialmente em instituições de saúde da gestão pública. Desse modo, o ultrassom à beira do leito é mais do que mera inovação tecnológica assistencial^(13)^, considerando o seu potencial no auxílio à gestão do cuidado.

Para o profissional usuário do POCUS, alguns benefícios também foram reconhecidos pela amostra, especialmente no que refere à tomada de decisões e autonomia do enfermeiro. Esses incrementos relacionaram-se à maior capacidade de argumentação, raciocínio clínico e fundamentação em dados. Assim, a tomada de decisões e escolhas do profissional seriam mais precisas, assertivas e resolutivas, refletindo na sua autonomia perante a equipe multiprofissional.

Especificamente no contexto dos cuidados críticos, uma avaliação rápida e dinâmica torna se essencial para intervenções imediatas. Portanto, a incorporação do POCUS nesses cenários pelo enfermeiro consegue promover maior robustez, segurança e eficácia para sistematizar o cuidado^(13)^.

No entanto, o uso do POCUS por enfermeiros é um objeto em disputa, ao tensionar interesses relacionados ao uso hegemônico do ultrassom à beira do leito por médicos^(5,41)^. Assim como o estetoscópio e o exame físico não são privativos dessa profissão, já sendo amplamente difundidos em outras profissões na área da saúde, o ultrassom à beira do leito também não pode ser restrito à medicina, visto que as pesquisas em saúde e resoluções já mostram o benefício e utilização desse recurso em cada profissão.

Dessa maneira, o uso do POCUS reflete questões mais amplas sobre a divisão de responsabilidades e autoridade entre diferentes profissões da área da saúde, bem como sobre a evolução dos papéis profissionais em resposta às mudanças na tecnologia e nas necessidades dos pacientes. Assim como na enfermagem, o emprego desse instrumento é recente por outros profissionais, tais como fisioterapeutas^(42)^, fonoaudiólogos^(43)^ e nutricionistas^(44)^. Por ser recente, o conhecimento de seus benefícios para a prática assistencial desses profissionais carece de aprofundamento.

Os enfermeiros também apontaram facilidades e dificuldades que condicionam a utilização do POCUS em seu cotidiano assistencial. A disponibilidade do aparelho de ultrassonografia para uso à beira do leito foi a facilidade mais frequente no cenário estudado, já que a presença do aparelho no ambiente de cuidados é um relevante aspecto que condiciona o seu uso.

A oferta de aparelhos de ultrassonografia nos hospitais depende de diferentes fatores, incluindo as políticas e recursos específicos de cada instituição de saúde. Entende-se que a modernização das interfaces dos aparelhos de ultrassonografia^(41)^, cada vez mais portáteis, pode facilitar a distribuição e o acesso do item em diferentes realidades e instituições.

Por fim, como dificuldades associadas ao uso do POCUS na realidade analisada, foram apontadas resistências de outros profissionais da equipe de saúde relacionadas à utilização do ultrassom pelo profissional enfermeiro; a necessidade de escalas bem dimensionadas para que o uso da ferramenta seja possível; e o fato de demandar do operador a familiarização com o equipamento, técnicas e configurações.

As resistências da equipe multiprofissional ao uso do ultrassom pelo enfermeiro podem revelar aspectos específicos da cultura organizacional (médico-centrada) da instituição estudada. Já as questões relativas ao dimensionamento de pessoal, que afetam o tempo de assistência do enfermeiro à beira do leito, também são vistas como barreira para a utilização do POCUS em outro estudo na realidade brasileira^(13)^. Além disso, as dificuldades técnico operacionais mencionadas pelos enfermeiros participantes alinham-se com achados de outro estudo^(33)^, sobretudo aquelas relacionadas à nitidez da imagem e às condições e colaboração do paciente avaliado.

### Limitações do estudo

O presente estudo apresentou limitações ligadas ao cenário de coleta de dados único e ao tamanho reduzido da amostra, determinado pelo número de enfermeiros habilitados em POCUS na instituição. Outro aspecto foi o fato de a legislação sobre o uso do POCUS ser recente no país, carecendo de publicações para aprofundar a discussão.

### Contribuições para a prática

Este estudo ajuda a ampliar o conhecimento sobre uma prática avançada em enfermagem. Ele mostra que o POCUS é uma ferramenta relevante para os cuidados do enfermeiro, principalmente em unidades de urgência e emergência ou de terapia intensiva. Os dados apontam procedimentos e avaliações que esse recurso pode apoiar, reforçando sua usabilidade na prática profissional do enfermeiro.

## CONSIDERAÇÕES FINAIS

O presente estudo, com base na percepção de enfermeiros que atuam em unidades de urgência, emergência e cuidados intensivos, descreveu a usabilidade do POCUS e identificou as intervenções apoiadas por essa tecnologia em cenários de cuidados críticos. Ficou evidenciado que o ultrassom à beira do leito se destaca como inovação e potencialidade para auxiliar os cuidados do enfermeiro.

Os enfermeiros participantes percebem o POCUS como uma ferramenta significativa para a assistência de enfermagem, tanto em unidades de emergência quanto de terapia intensiva. Nesse contexto, ele melhora a tomada de decisões e autonomia diante de procedimentos e cuidados prestados aos pacientes, permitindo que o profissional realize avaliação antes, no decorrer e depois de uma intervenção de enfermagem.

Ficou demonstrado o uso do POCUS para procedimentos relacionados com a assistência de enfermagem, com destaque para a punção venosa periférica, punção arterial e avaliação e/ou procedimentos vesicais. Também foram mencionados outros procedimentos, tais como a avaliação cardiovascular, avaliação e realização de procedimentos gastroenterais, avaliação pulmonar, verificação de posicionamento de tubo orotraqueal e avaliação de pressão intracraniana. Assim, ressalta-se sua usabilidade no cotidiano assistencial de enfermeiros.

Portanto, partindo do pressuposto de que o POCUS detém potencial para transformar a prática da enfermagem, recomenda-se a realização de estudos que explorem sua usabilidade e efetividade em diferentes áreas de cuidados da enfermagem e que identifiquem suas contribuições para a segurança do paciente, sobretudo mediante registros assistenciais. É necessário, sobretudo, que o investimento institucional e governamental seja fortalecido, visando ampliar a formação e treinamento dos enfermeiros para o emprego desse instrumento, de modo que os benefícios de seu uso estejam disponíveis para todos de forma plena e segura.
